# Rheumatoid arthritis presenting by combined pulmonary fibrosis and emphysema with clubbing and pulmonary hypertension: A rare case report

**DOI:** 10.1002/ccr3.9535

**Published:** 2024-10-28

**Authors:** Reza Basiri, Javad Davoodian, Amir Baniasad

**Affiliations:** ^1^ Lung Disease Research Center Mashhad University of Medical Sciences Mashhad Iran; ^2^ Faculty of Veterinary Medicine, Urmia Branch Islamic Azad University Urmia Iran

**Keywords:** clubbing, combined pulmonary fibrosis and emphysema, pulmonary hypertension, rheumatoid arthritis

## Abstract

A 60‐year‐old male smoker came with dyspnea. A rare condition known as combined pulmonary fibrosis and emphysema (CPFE) was observed on a chest CT scan. Decreased DLCO in combination with normal FVC can help diagnose CPFE. Concomitant connective tissue diseases, including rheumatoid arthritis, should be checked in CPFE patients.

## 1 INTRODUCTION

Combined pulmonary fibrosis and emphysema (CPFE) is a rare condition in which emphysema and fibrosis are simultaneously present in the lungs of patients.^1^ This disease usually occurs in people with a history of smoking and is manifested by shortness of breath, cough, and distinct radiological changes.^2^ The diagnosis of this condition is based on the findings of the patient's chest CT scan.^1^, ^2^

Rheumatoid arthritis (RA) is a systemic inflammatory disease characterized by the inflammation and destruction of the joints.^3^ It is also associated with the involvement of extraarticular organs, including the lungs, due to systemic inflammation.^4^ The most common pulmonary manifestation of RA is ILD, and ILD signs are reported in 30%–60% of RA patients.^5^ The prevalence of CPFE in RA patients with ILD and RA patients with lung cancer reported 13.3% and 40.2%, respectively.^3^, ^4^

Despite the progress made, treating CPFE patients is still challenging. The treatment of these patients is complicated, and due to the simultaneous occurrence of emphysema, fibrosis, and pulmonary hypertension in most patients and the effect of each of these pathologies on the prognosis of the disease, appropriate treatment for all these complications should be prescribed for the patient.^2^

The following case represents a case of CPFE in the context of RA with clubbing and pulmonary hypertension.

## 2 CASE HISTORY/EXAMINATION

A 60‐year‐old male with a 50‐pack‐year history of smoking and a long history of dyspnea came to our tertiary hospital with a complaint of dyspnea. The patient had shortness of breath at rest and an exacerbating productive cough that had been present for a month. He had no fever or dyspnea, and he did not wake up at night due to dyspnea. The patient did not have any evidence of gastroesophageal reflux disease.

**FIGURE 1:**
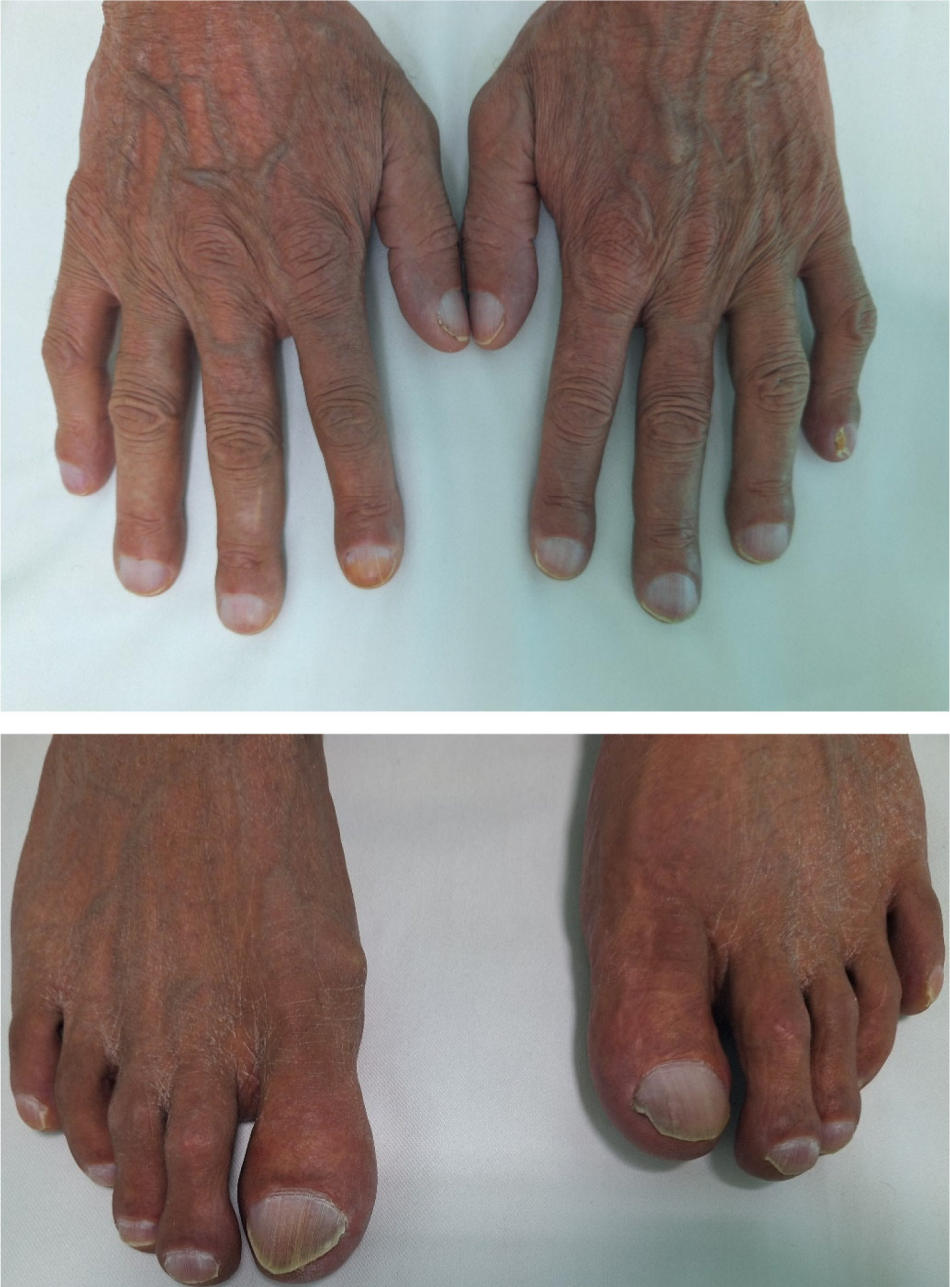
Clubbing in the fingers and toes of the patient.

The patient's job was as a fishing boat captain until 4 years ago, and he had a history of a transient ischemic attack (TIA) 6 years ago. He irregularly used albuterol and ipratropium bromide spray, which was administered by his physician 4 years ago for a short period of dyspnea without performing spirometry or any other investigation. The patient experienced a weight loss of 52 kg (from 100 kg to 48 kg) in the last 2 years. The patient had no history of occupational exposure to risk factors for pulmonary diseases. The patient had no positive first or second‐degree family history for similar pulmonary diseases. At the first visit to our center, the patient's oxygen saturation was 86%, which reached 94% after the administration of 6 L/min oxygen therapy through a nasal cannula. The patient's blood pressure was 100/80 mmHg, respiration rate was 24 per minute, and body temperature was 36.7. In the examination, the patient had cyanosis in the lips. Lung auscultation had crackles in the lung bases. There was clubbing in the fingers and toes (Figure 1). The patient's laboratory findings are shown in Table 1.

**TABLE 1:** Primary laboratory findings of the patient.

Investigations	Results	Reference range
CRP (mg/dL)	21.4	0–6
ESR (mm/h)	77	0–15
WBC (×103/uL)	12.2	4–10
RBC (×106/uL)	5.73	4.5–5.5
Hb (gr/dL)	14.5	13–17
Hct (%)	47.5	40–50
Platelet (×109/uL)	323	150–450
Absolute neutrophil count (×103/uL)	8.67	–
Lymphocyte (×103/uL)	2.79	–
PCR assays for SARS‐COV‐2	positive	–
Venous blood gas (VBG)		
PH	7.40	7.35–7.45
Bicarbonate (mmol/L)	20.7	22–28
pCO2 (mmHg)	32.5	36–46

Abbreviations: CRP, C‐reactive protein; ESR, erythrocyte sedimentation rate; hemoglobin; Hct, hematocrit; PCR, polymerase chain reaction; RBC, red blood cells; SARS‐COV‐2, severe acute respiratory syndrome coronavirus 2; WBC, white blood cells.

## 3 DIFFERENTIAL DIAGNOSIS, INVESTIGATIONS, AND TREATMENT

**FIGURE 2:**
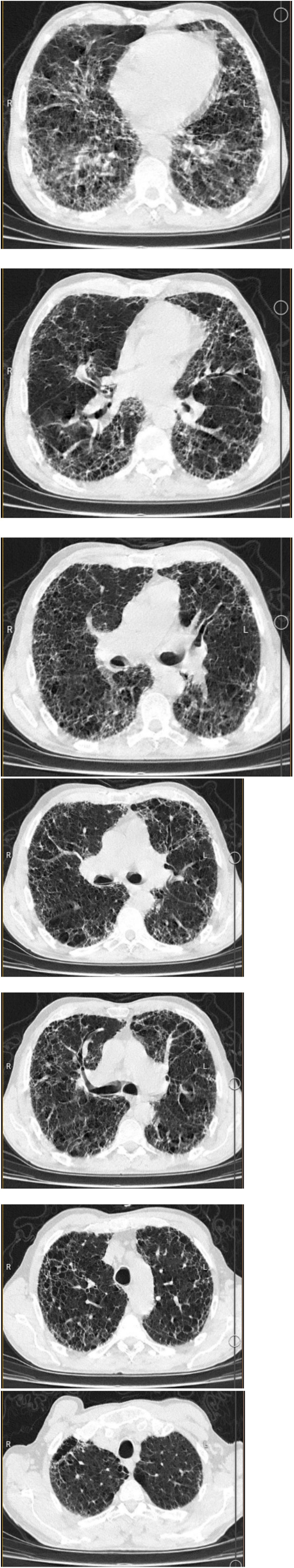
Centrilobular and paraseptal emphysema (preferably in the upper lobe) with fibrotic changes and increased thickness of inter and intralobular septa in the peripheral regions, preferably in the lower lobe of the lung. Tractional bronchiectasis secondary to fibrotic changes is present in the lung parenchyma.

In chest computed tomography (CT), the patient had centrilobular and paraseptal emphysema with fibrotic changes and increased thickness of inter and intralobular septa in the peripheral regions, preferably in the lower lobe of the lung (Figure 2). Considering the simultaneous changes of emphysema and pulmonary fibrosis, the diagnosis of the CPFE was confirmed.

Treatment with methylprednisolone (40 mg every 12 h), ampicillin‐sulbactam (3 g every 6 h), supplemental oxygen, bronchodilator (including albuterol and ipratropium), and inhaled corticosteroids (Fluticasone propionate) was started for the patient. Considering the lung fibrosis in the patient's CT scan, treatment with nintedinib (150 mg every 12 h) was also started for the patient.

One week after admission, pulmonary function tests (PFTs) were performed for the patient (Table 2). In the spirometry, the patient's forced expiratory volume in 1 s (FEV1) (72% of expected) and forced expiratory flow between 25% and 75% of FVC (FEF25‐75) decreased (58% of expected). Forced vital capacity (FVC) was in the normal range in our patient (89% of the expected value). The FEV1/FVC ratio was reduced in our patient (64%). Due to the patient's lack of ability and cooperation, we could not measure the patient's lung diffusion for carbon monoxide (DLCO) and body plethysmography.

**TABLE 2:** Spirometer findings of the patient during hospitalization.

Parameter	Reference average (reference ranges)	Pre‐bronchodilators (% of reference average)	Post‐bronchodilators (% of reference average)
FVC (liters)	4.29 (3.68–4.90)	3.80 (89)	3.86 (90)
FEV1 (liters)	3.40 (2.89–3.91)	2.45 (72)	2.58 (76)
PEF (liters/seconds)	8.46 (7.25–9.67)	7.03 (83)	7.36 (87)
FEV1/FVC (%)	77 (70–84)	64 (83)	67 (87)
FEF25‐75 (liters/seconds)	3.62 (2.58–4.66)	2.12 (58)	2.32 (64)

Abbeviations: FVC, forced vital capacity; FEV1, forced expiratory volume; FEF25‐75, forced expiratory flow between 25% and 75% of FVC; PEF, peak expiratory flow.

In our patient's echocardiography, left ventricular size was normal, and global systolic function was preserved (left ventricular ejection fraction [LVEF] of 50%–55%). The patient had Right ventricle enlargement with moderate to severe systolic dysfunction and pulmonary hypertension (systolic pulmonary artery pressure [SPAP] of 50 mmHg).

During the follow‐up visit, the patient was referred for body plethysmography, the results of which are shown in Table 3. The patient had normal FEV1 (Forced expiratory volume), FVC (Forced vital capacity), and FEV1/FVC ratio, but DLCO was markedly decreased (18% of the predicted value).

**TABLE 3:** Body plethysmography findings of the patient 3 months after hospitalization.

Parameter	Predicted	Actual	Actual/predicted
FVC (liters)	4.08	3.52	86%
FEV1 (liters)	3.24	2.57	79%
PEF (liters/seconds)	8.25	8.96	109%
FEV1/FVC (%)	77	73	95%
FEF25‐75 (liters/seconds)	3.53	2.21	63%
Raw total (kPa/ liters/seconds)	<0.30	0.16	55%
sRaw total (kPa/ seconds)	<1.18	0.79	67%
TLC (liters)	6.75	6.89	102%
TGV (liters)	3.50	4.81	137%
RV (liters)	2.33	3.25	139%
RV/TLC (%)	37	47	129%
TGV/TLC (%)	56	70	124
DLCO (mmol/min/kPa)	9.30	1.65	18%
KCO (mmol/min/kPa/liters)	1.40	0.25	18%
VA (liters)	–	6.65	–

Abbreviations: DLCO, diffusing capacity of the lung for carbon monoxide; FEF25‐75, Forced expiratory flow between 25% and 75% of FVC; FEV1, forced expiratory volume; FVC, forced vital capacity; KCO, transfer coefficient of carbon monoxide; PEF, peak expiratory flow; Raw, airway resistance; RV, residual volume; sRaw, specific airway resistance; TGV, THORACIC gas volume; TLC, total lung capacity; VA, alveolar volume.

Considering the presence of arthralgia and morning stiffness and the association between CPFE and connective tissue diseases (CTDs), the laboratory test for the screening of CTDs was performed for the patient (Table 4). The patient had positive results for rheumatoid factor (RF) and Anti‐cyclic citrullinated peptide (anti‐CCP). So, the patient was referred to a rheumatologist, and the diagnosis of RA was confirmed for the patient. The methylprednisolone (1 g once daily) for 3 days was administered to the patient. The treatment with hydroxychloroquine (200 mg once daily) and prednisolone (25 mg once daily) was started for the patient. The prednisone dose was planned to taper until 10 mg once daily in subsequent visits according to the response to the treatment.

**TABLE 4:** Rheumatologic laboratory tests results of the patient.

Investigations	Results	Reference range
Rheumatologic tests		
ANA (IU/mL)	9.2	<23
Anti‐CCP (AU/mL)	5	<12
RF (U/mL)	8	<20

Abbeviations: ANA, antinuclear antibody; Anti‐CCP, anti‐cyclic citrullinated peptide; RF, rheumatoid factor.

## 4 OUTCOME AND FOLLOW‐UP

The patient was discharged with the mentioned treatment. He was advised to visit a pulmonologist every 3 months and a rheumatologist every month. The patient became a candidate for lung transplantation. The smoking cessation was advised for the patient.

## 5 DISCUSSION

The most accurate diagnostic test for CPFE is a chest CT scan; the simultaneous presence of fibrosis in the lower lobes and emphysema in the upper lobes is crucial in confirming the diagnosis of patients.^1^, ^6^ Emphysematous changes in the upper lobe can be centrilobular and paraseptal, and fibrotic changes include honeycombing and bronchiectasis.^1^

Among people who have CTDs and CPFE at the same time, the prevalence of RA is 53%, and as our patients, it is essential to screen CPFE patients for concomitant CTDs.^7^ In the study of Bonilla Hernán et al., the prevalence of CPFE in RA patients with ILD was reported at 13.3%, and all of these patients were male, smokers, and more than 55 years old.^3^ This phenotype, which was compatible with our patient, can lead to the more accelerated diagnosis of these patients.

The exact prevalence of CPFE is unknown. The prevalence of this disease in asymptomatic male smokers is estimated at 3.1%.^8^ The relationship between this disease and smoking has been previously reported. Patients who continued to smoke had a faster progression of fibrosis. About 7% of asymptomatic CPFE patients develop lung cancer during follow‐up. The prevalence of CPFE in RA patients with lung cancer was reported to be 40.2%, and the presence of CPFE was associated with an increased risk of lung cancer‐related mortality, so periodic examination of these patients with CT reduces complications.^1^, ^4^, ^8^ Our patient was also a heavy smoker who was advised to stop smoking, but there was no evidence in favor of lung cancer in the CT scan.

Clubbing is present in idiopathic pulmonary fibrosis (IPF) and other forms of interstitial lung disease (ILD) and is associated with poor prognosis. Previous studies reported the presence of clubbing in CPFE patients.^1^, ^9^ Many lung diseases can be associated with clubbing, but the association of clubbing with emphysema is not expected.^10^ So, the concomitant presence of clubbing and emphysema suggests the presence of other pathologies like cancer or fibrosis.

Right ventricle enlargement and pulmonary hypertension in our patient can be explained in the context of the patient's restrictive lung disease.^1^

PFT can also help diagnose CPFE patients. Due to the accompanying obstructive and restrictive lung disease, patients with IPF and emphysema usually present with normal or increased FVC along with a decrease in the DLCO.^11^ PFT findings of CPFE patients usually indicate a relatively preserved function with a decrease in DLCO.^12^ Our patient had decreased Forced expiratory flow between 25% and 75% of FVC (FEF25‐75) and increased Residual volume (RV) compatible with obstructive pulmonary disease, but FVC and FEV1/FVC were in the normal range. The DLCO and KCO (DLCO/alveolar volume) were prominently decreased, and overall, this pattern in PFT is compatible with CPFE.

A significant percentage of CPFE patients may also have an underlying autoimmune disorder, in which case the prognosis is usually more favorable.^13^, ^14^ However, the prognosis of a combination of CTDs and CPFE is worse than that of a combination of CTDs and ILD.^15^ In our patient, antinuclear antibody (ANA), RF, and Anti‐cyclic citrullinated peptide (anti‐CCP) were checked, and according to the results, the diagnosis of RA was established for the patient.

New biomarkers, including club cell secretory protein (CC16), surfactant protein D (SP‐D), and Krebs von den Lungen 6 (KL‐6), were reported to provide helpful information for diagnosis and prediction of prognosis in CPFE.^16^, ^17^, ^18^ These biomarkers play a role in the pathogenesis of pulmonary fibrosis.^16^, ^17^, ^18^ However, considering these markers are too expensive and we do not have access to them, we could not measure them.

The RA treatment, including glucocorticoid and nintedanib, was prescribed for our patient. Nintedanib is a tyrosine kinase inhibitor that reduces disease progression in IPF patients by reducing the rate of decline in FVC.^11^, ^19^ Although treatment with nintedanib was approved for IPF patients in previous studies, the administration of nintedanib for patients with concomitant emphysema and fibrosis (like CPFE) is still a challenge.^11^, ^19^ Some previous studies reported a similar effect of nintedanib on FVC decline in IPF patients with and without emphysema.^11^, ^19^

## 6 CONCLUSION

Despite the significant progress in treating emphysema and ILD patients, treating CPFE patients is still a challenge, and more studies are needed in this field. Screening for CTDs, including RA, is necessary in CPFE patients for an accelerated diagnosis of patients with a higher risk of complications and choosing a better therapeutic approach. Nintedanib can help treat these patients. The presence of clubbing in patients with emphysema can suggest the diagnosis of CPFE, and the Chest CT of these patients should be evaluated more carefully due to the presence of evidence of fibrosis. PFT findings, including decreased DLCO combined with normal FVC and increased RV, can help diagnose CPFE.

## AUTHOR CONTRIBUTIONS

**Reza Basiri:** Conceptualization; investigation; project administration; supervision; validation; writing – review and editing. **Javad Davoodian:** Investigation; writing – review and editing. **Amir Baniasad:** Conceptualization; investigation; methodology; project administration; writing – original draft; writing – review and editing.

## FUNDING INFORMATION

There is no external funding source for this case report.

## CONFLICT OF INTEREST STATEMENT

There is no conflict of interest.

## ETHICS STATEMENT

The study was reviewed and approved by the ethics committee of Mashhad University of Medical Sciences (ethic code: IR.MUMS.REC.1403.076).

## CONSENT

Written informed consent was obtained from the patient to publish this report in accordance with the journal's patient consent policy.

## Data Availability

The data that support the findings of this study are available from the corresponding author upon reasonable request.
